# Effects of environmentally relevant ibuprofen and valproic acid exposure on zebrafish behavior

**DOI:** 10.17305/bb.2026.14030

**Published:** 2026-04-21

**Authors:** Ionut-Alexandru Chelaru, Ramona-Alexandra Ciausu, Alexandra Savuca, Camelia Ureche, Mircea Nicusor Nicoara, Alin Stelian Ciobica, Gabriel-Andrei Andronic, Rares-Mircea Ambrosie, Dorel Ureche

**Affiliations:** 1 Doctoral School of Geosciences, Faculty of Geography and Geology, “Alexandru Ioan Cuza” University of Iasi, Iasi, Romania; 2 Department of Biology, Faculty of Sciences, “Vasile Alecsandri” University of Bacau, Bacau, Romania; 3 Department of Biology, Faculty of Biology, “Alexandru Ioan Cuza” University of Iasi, Iasi, Romania; 4 “Ioan Haulica” Institute, Apollonia University, Iasi, Romania; 5 Department of Biomedical Research, “Olga Necrasov” Center, Romanian Academy, Iasi, Romania; 6 Multidisciplinary Medical Research and Development Platform in the North-East Region (CENEMED), “Grigore T. Popa” University of Medicine and Pharmacy of Iasi, Iasi, Romania; 7 Academy of Romanian Scientists, Bucharest, Romania; 8 Department of Mathematics and Informatics, Faculty of Sciences, “Vasile Alecsandri” University of Bacau, Bacau, Romania

**Keywords:** Zebrafish, pharmaceuticals, behavior, ecotoxicology

## Abstract

Active pharmaceutical ingredients (APIs) are increasingly entering aquatic environments due to human and veterinary use, wastewater discharges, and inadequate waste containment, raising significant concerns for both ecosystems and human health. Ibuprofen (Ibu) and valproic acid are among the pharmaceuticals detected in surface waters, primarily due to incomplete metabolism and the limited removal efficiency of conventional wastewater treatment systems. Ibu, readily available over the counter, is frequently found in high concentrations, while valproic acid, which is available only by prescription, is detected less often, likely reflecting its more restricted use. This study employed the established behavioral ecotoxicology model, *Danio rerio*, to investigate the effects of environmentally relevant concentrations of Ibu (20 µg L^−1^) and valproic acid (3 µg L^−1^) on zebrafish (8 months old) after 96 h of exposure. Both compounds influenced locomotor and anxiety-related endpoints, with changes in social preference primarily associated with valproic acid exposure. Single compound exposures resulted in reduced total distance traveled and average velocity, while combined exposure did not differ from the control group, indicating no additive locomotor impairment. Inactivity duration decreased in both individual treatments, most significantly with valproic acid, whereas the mixture produced no significant effect. Only Ibu reduced counterclockwise rotations, suggesting a mild anxiolytic-like response. Given the ecological importance of social cohesion and locomotor performance in predator avoidance, foraging, and reproduction, such behavioral disruptions may compromise population stability. These findings highlight the necessity of integrating behavioral endpoints and considerations of mixture toxicity into ecological risk assessments of pharmaceutical contaminants in aquatic systems.

## Introduction

Active pharmaceutical ingredients (APIs) enter the natural environment at various stages of their life cycle, including manufacturing, consumer use, and disposal. Rapid urbanization and the expansion of pharmaceutical production have intensified their release into aquatic ecosystems, raising significant ecological [1] and human health concerns [2–5].

After administration, pharmaceuticals such as Ibu and valproic acid (VPA) are only partially metabolized in the human body, with a portion excreted in urine and feces [5]. Two major sources of pharmaceutical consumption are human and veterinary medicine [6]. In the human sector, pharmaceutical residues primarily originate from hospitals and household wastewater [5, 7]. In veterinary contexts, animal waste from livestock farms ideally is collected on specially designed platforms and subsequently neutralized [8, 9].

Unfortunately, many livestock operations lack dedicated containment structures for waste storage, leading to leachate formation [10, 11], which can be facilitated by precipitation. This leachate may reach surface and groundwater [12, 13] and, critically, drinking water supplies [14, 15]. The issue is exacerbated by conventional water and wastewater treatment systems that are not designed to remove pharmaceutical compounds, resulting in widespread detection of drug residues in aquatic ecosystems around the world [16–19].

Given the prevalence of pharmaceutical pollutants in natural aquatic environments, this study focuses on two representative compounds: the anti-inflammatory drug Ibu and the antiepileptic agent VPA. These substances were selected due to their high consumption rates, frequent detection in surface waters, and documented potential to disrupt physiological and behavioral processes in aquatic organisms. The study employs concentrations commonly reported in surface waters to enhance ecological relevance and discuss the potential risks associated with short-term exposure (96 h).

To contextualize their ecological relevance, the following subsection provides a detailed description of Ibu and VPA, including their pharmacological roles, environmental behavior, and typical concentrations detected in natural water bodies.

Ibu is a nonsteroidal anti-inflammatory drug (NSAID) widely used for its analgesic, antipyretic, and anti-inflammatory effects [20–22]. Its pharmacological activity stems from the non-selective inhibition of cyclooxygenase-1 (COX-1) and cyclooxygenase-2 (COX-2) enzymes, which are critical for prostaglandin synthesis [23, 24]. Compared to other NSAIDs [22, 25], Ibu has moderate anti-inflammatory potency. Its high frequency of use and elevated concentrations in the environment are likely due to its availability as an over-the-counter medication [26–28].

Monitoring studies have reported significant concentrations of Ibu globally. For example, levels in wastewater from Pakistan ranged from 703–1673 µg L^−1^ [29], while concentrations in the Mankyung River (South Korea) [30] to were reported at 414 ng L^−1^ and 1417 ng L^−1^ in surface waters [19]. In European groundwater, Ibu concentrations ranged from 3 to 395 ng L^−1^ [31], and it has been detected in drinking water at levels as low as 3 ng L^−1^ [32]. Rainsford (2009) described Ibu as having a safety profile comparable to that of paracetamol, particularly when administered within therapeutic limits [33]; however this safety does not extend to the environment. Due to its high consumption, incomplete metabolic degradation, and limited removal during wastewater treatment, Ibu enters natural waters, posing risks to aquatic organisms even at low concentrations. Its persistence also facilitates leaching into groundwater, potentially reintroducing it into drinking water supplies and perpetuating a cycle of environmental and human exposure.

VPA, a widely prescribed antiepileptic drug, is used for various seizure types and epileptic syndromes [34]. Although primarily designed for therapeutic applications, VPA has emerged as a significant environmental pollutant. It enters aquatic systems through various pathways, including wastewater treatment plant (WWTP) discharges, excretion of unmetabolized compounds and metabolites, agricultural runoff, and improper disposal of medicines [35–37]. Due to its teratogenic and neurodevelopmental risks, VPA is strictly regulated and available only by prescription [38], suggesting its presence in aquatic environments should theoretically be low. Nonetheless, its detection in water bodies indicates that prescription drugs can reach these ecosystems.

Conventional wastewater treatment technologies were not originally designed to remove pharmaceuticals, resulting in their detection at minute concentrations (ng L^−1^ to a few mg L^−1^) in surface waters [36]. VPA has been found in wastewater influent and effluent at nanogram-per-liter concentrations, demonstrating resistance to conventional treatment processes. For instance, measured levels of VPA at the Back River WWTP in the USA were reported at 130 ng L^−1^ [39], while environmental monitoring in Nanjing, China, revealed concentrations of approximately 0.834 µg L^−1^, indicating its persistence and the presence of pharmaceutical residues in natural waters [40].

Given its widespread clinical use and confirmed presence in aquatic environments, VPA is recognized as an emerging environmental pollutant, capable of affecting aquatic organisms at environmentally relevant concentrations. According to findings by Lana et al. (2016), Ibu may reduce total VPA concentrations by displacing it from plasma proteins, thereby increasing free VPA and accelerating its metabolism [41]. This interaction suggests potential antagonistic effects, warranting further examination of their dynamics in real environmental conditions. To enhance awareness of the negative impacts of these emerging pollutants, research has focused on laboratory modeling using established model organisms, such as zebrafish (*Danio rerio*). The use of *Danio rerio* in pharmaceutical pollution studies is justified [42] due to its rapid reproduction, ease of maintenance, and high genetic similarity to humans [43–45], making it a relevant subject for ecotoxicology and human disease studies [46]. Its early developmental transparency [47], well-characterized behavior, and compatibility with high-throughput toxicology make it particularly valuable for environmental risk assessment [48–50].

As highlighted by Ortúzar et al. (2022), advanced biological treatment technologies, such as modified conventional activated sludge, aerobic granular systems, moving bed bioreactors, membrane bioreactors, and Anammox processes, play a central role in enhancing wastewater purification [51]. Their schematic overview provides a useful framework for understanding how these systems operate and how by-products, such as sewage sludge, may contribute to the transfer of pharmaceuticals to the environment (Figure 1).

**Figure 1.**
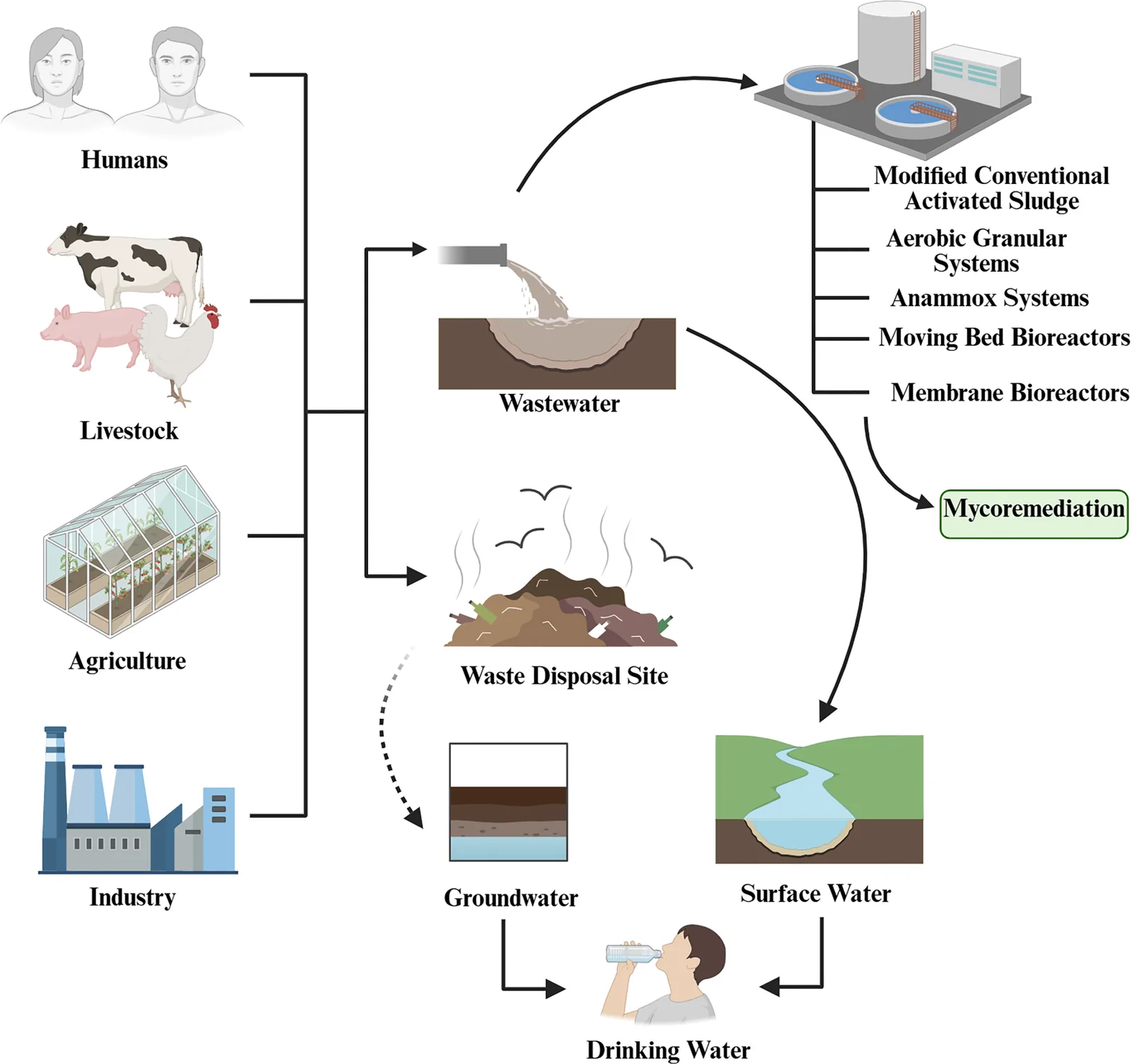
**Environmental pathways of pharmaceutical contamination and biological treatment approaches in aquatic systems.** Schematic representation of the major sources and transport routes of pharmaceutical contaminants in the environment. Pharmaceuticals originating from human use, livestock production, agriculture, and industrial activities may enter wastewater streams or waste disposal sites, from which they can subsequently reach surface water and groundwater and ultimately contribute to drinking water contamination. The figure also summarizes advanced biological wastewater treatment technologies that improve pharmaceutical removal, including modified conventional activated sludge, aerobic granular systems, anaerobic ammonium oxidation (Anammox) systems, moving bed bioreactors, membrane bioreactors, and mycoremediation. Adapted from Ortúzar et al. [51]; *created in BioRender.*

## Materials and methods

### Ethical statement

All husbandry and experimental procedures were conducted in full compliance with current Romanian legislation and received ethical approval from the Ethics Committee of the Faculty of Biology, “Alexandru Ioan Cuza” University of Iasi, Romania (Approval No. 1273/19.05.2023). The study adhered to national and institutional guidelines for the care and use of laboratory animals and complied with Directive 2010/63/EU on the protection of animals used for scientific purposes, which establishes standards for housing, husbandry, environmental enrichment, and monitoring of animal welfare. Fish were euthanized by individually immersing them in cold water combined with ice until opercular movement ceased, in accordance with American Veterinary Medical Association (AVMA) Guidelines [52].

### Test substances

Liquid (400 mg 100 mL^−1^) and VPA tablets (500 mg) were obtained from licensed local pharmacies through the institution. Fresh stock solutions of Ibu (100 mg L^−1^) and VPA (100 mg L^−1^) were prepared *de novo* by dissolving the respective compounds in distilled water. Ibu, being highly water-soluble, was dissolved directly. VPA tablets (500 mg) were finely crushed using an agar mortar and pestle, with the amount of powder calculated based on the active VPA content indicated in the prescription. After weighing, the powder was dispersed in distilled water and homogenized using a vortex mixer followed by an ultrasonic bath to ensure complete dispersion. The resulting stock solution was then vacuum-filtered to remove any undissolved excipients or tablet coating residues.

### Zebrafish maintenance

Adult wild-type AB strain zebrafish (*Danio rerio*), aged 8 months, with a mean body mass of 0.73 ± 0.1 g and a mean standard length of 41 ± 3 mm, were sourced from a certified breeder in Iasi, Romania, adhering to national animal welfare regulations. The zebrafish were acclimated to laboratory conditions for 14 days in a 92 L aquarium (60 × 30 × 45 cm) prior to behavioral testing. They were subsequently transferred to smaller 10 L test tanks (30 × 17 × 20 cm) for an additional 3 days to facilitate further adaptation. Following this acclimation period, fish were randomly assigned to four treatment groups: Control, Ibu, VPA, and Ibu+VPA (*n* ═ 5/group; 3:2 male:female ratio). After 96 h of exposure, individual fish were evaluated in a randomized sequence according to housing conditions, starting with the control group until all fish had been tested.

During both the acclimation and testing phases, the fish were housed in tanks equipped with continuous oxygenation and filled with dechlorinated water, which was fully renewed daily. Water quality parameters were maintained consistently, with a temperature of 26 ± 1 ^∘^C, pH 7.5, dissolved oxygen at 7.1 ± 0.1 mg L^−1^, ammonia levels below 0.004 ppm, and conductivity of 500 µS. Behavioral assessments were conducted in a dedicated testing room between 9:00 AM and 5:00 PM, with each fish undergoing individual behavioral assays. The light cycle was set to 14 h of light and 10 h of darkness, while fish were fed twice daily with commercial dry food (e.g., TetraMin™ flakes) at a daily ration of approximately 2% of their body weight, with environmental parameters maintained according to previously established protocols [53, 54].

### Experimental design

Zebrafish were categorized into four groups: a control group that experienced identical housing and environmental conditions as the treated groups but did not receive any substance administration, and three experimental groups receiving Ibu, VPA, and a combination of both (Ibu+VPA). The exposure concentrations were set at 20 µg L^−1^ for Ibu and 3 µg L^−1^ for VPA, prepared from stock solutions, with equivalent concentrations utilized in the Ibu+VPA mixture. These concentrations were selected based on values reported in environmental monitoring studies and previous ecotoxicological research utilizing environmentally relevant doses derived from WWTP effluent measurements. According to Borova et al. (2014), VPA was detected at concentrations ranging from 202.8–3008 ng L^−1^, while Ibu levels were reported between 0.05 and 24.6 µg L^−1^ in WTP influents and effluents [55, 56].

To evaluate the individual and combined effects of Ibu and VPA, the Social Preference Test was employed. Behavioral endpoints were assessed after a 96-hour exposure to the pharmaceuticals using EthoVision XT 17 video tracking software (Noldus Information Technology, Wageningen, The Netherlands) for a 4-minute assay per trial. The behavioral parameters evaluated included total distance traveled (DT), average velocity (AV), immobility duration (ID), counter-clockwise rotations (CCW), time spent in the start arm (SA), time spent in the decision zone (DZ), time spent in the divided left arm (LA), and time spent in the right arm (RA). All parameters were calculated based on changes in the animal’s center point position, with movement defined by a velocity threshold of 2 cm/s to 1.75 cm/s. The center point was automatically detected by the software, with manual adjustments made as necessary before data acquisition commenced. Inactivity was defined as periods where the center point velocity fell below 20%. CCW rotations were quantified using EthoVision’s angular velocity algorithm, identifying full 360∘ turns around the animal’s center of mass, reported as frequency (number of events) per trial, requiring a minimum displacement of 2 cm and a 50∘ angular threshold. Time spent in each arm of the T-maze was quantified by calculating the total time (s) the fish remained within each predefined zone, based on EthoVision’s zone entry and zone exit timestamps. At the conclusion of the experiment, fish were euthanized following approved ethical procedures.

### Social preference test

The Social Preference Test was conducted using a T-shaped maze (Testing Arena) comprising four zones: Start Arm, Decision Zone, Divided Left Arm, and Right Arm (Figure 2). Each zebrafish was individually placed in the SA and tracked for the duration of a 4-min assay. This configuration enables the assessment of social decision-making under various treatment conditions, as the fish must choose between arms upon reaching the DZ of the maze. To evaluate social preference, three conspecifics were placed in a compartment adjacent to the Left Arm, separated from the maze by a transparent divider. Stimulus fish were matched in a 2:1 ratio of males to females and were similar in size to the focal fish used in the assay. The three stimulus fish were rotated between group trials to prevent repeated exposure to the same individuals, and all fish were handled in a manner that minimized stress and habituation. The test fish was introduced into the SA, with time spent in the Left Arm serving as an indicator of social behavior, supplemented by locomotor parameters such as DT, AV, ID, and CCW, recorded using EthoVision XT 17 software. Figure 2 illustrates the layout of the T-maze used in the behavioral assay, along with relevant structural annotations and designated zones.

**Figure 2.**
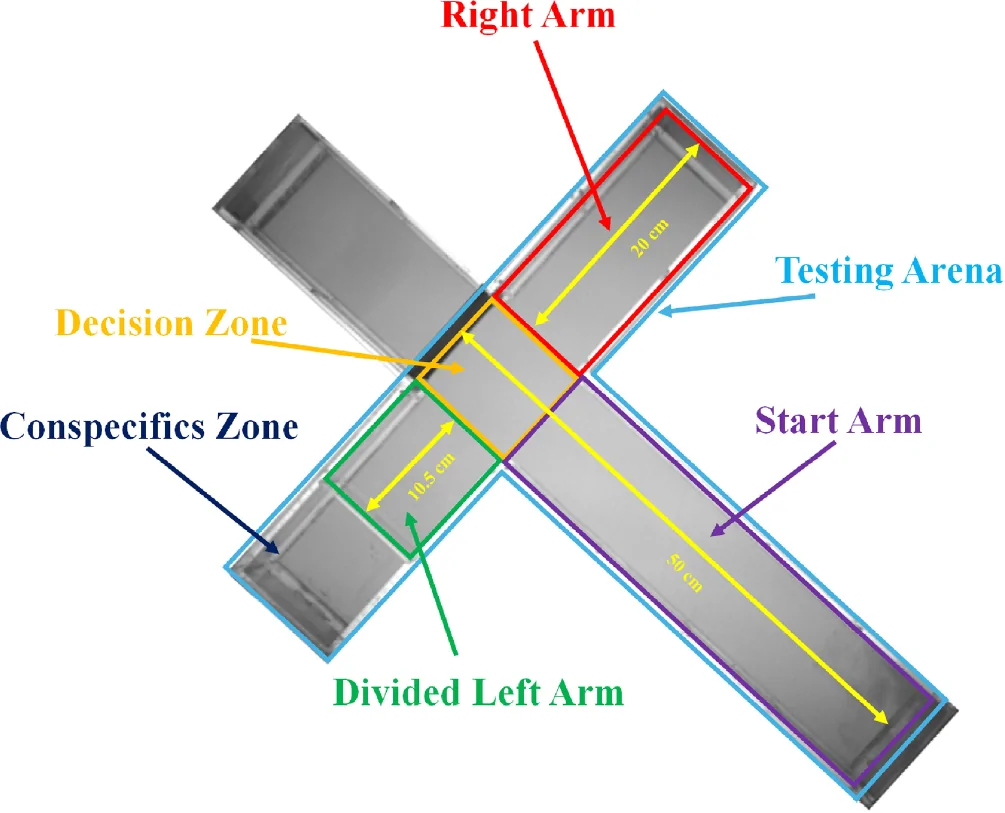
**T-shaped maze configuration used in the Social Preference Test.** The testing arena consisted of a start arm (25 cm), a central decision zone, a right arm (20 cm), and a divided left arm (10 cm) positioned adjacent to a separate conspecifics zone. During the 4-min assay, each focal zebrafish was introduced into the start arm and allowed to explore the maze freely; upon reaching the decision zone, the fish could choose between the right arm and the divided left arm, which was separated from three conspecific stimulus fish by a transparent partition. Time spent in the divided left arm was used as the primary indicator of social preference.

### Statistical analysis

Group comparisons were conducted using one-way analysis of variance (ANOVA) followed by Tukey’s Honestly Significant Difference (HSD) post hoc test. For statistical analyses, individual fish were treated as the experimental unit, consistent with behavioral toxicology studies where exposure is shared, but measurements are taken independently. Results are reported as mean ± standard deviation (SD) with 95% confidence intervals (CI), and statistical significance was established at *P* < 0.05. Letter-based groupings were employed to succinctly summarize multiple comparisons and simplify interpretation. Groups sharing at least one letter are not significantly different, while groups with no common letters differ significantly. This format facilitates a concise summary of multiple comparisons. All graphs and statistical analyses were generated using GraphPad Prism 10.4.1 (San Diego, CA, USA).

Pairwise associations between behavioral variables were examined using Pearson’s correlation coefficient (*r*). Correlations were calculated between each swimming parameter (DT, AV, CCW) and the time spent in each T-maze arm, utilizing individual fish as the unit of analysis. All variables were initially assessed for normality and linearity to confirm their suitability for Pearson’s method. The sample size (*n* ═ 20) for each correlation corresponded to the total number of fish with complete data for the two variables being compared. Correlation coefficients and their associated *P*-values were computed using the full dataset without pooling across treatments, allowing for the assessment of continuous relationships independent of group assignment. Statistical significance was evaluated at *α* ═ 0.05.

## Results

Social behavior was evaluated after 96 h of single and combined exposure to environmentally relevant concentrations of Ibu and VPA. Following exposure, each zebrafish underwent a social preference assay conducted in a T-shaped maze. An isolated compartment containing three untested conspecifics was positioned next to the divided left arm of the apparatus, separated from the test area by a transparent partition that permitted visual but not physical interaction. Each experimental fish was placed in the SA and tracked for a 4-min trial to assess its social preference behavior.

Analysis of the time spent in the SA of the T-maze revealed that both the Ibu-exposed group (*P* ═ 0.005, 95% CI [2.86, 16.83]) and the Ibu+VPA co-exposed group (*P* < 0.001, 95% CI [5.08, 19.05]) exhibited a significant reduction compared to the control group. Conversely, as illustrated in Figure 3A, the VPA-treated group did not differ significantly from the control group (_p_ > 0.05, 95% CI [−6.98, 6.98]). Furthermore, the VPA-treated group demonstrated increased time spent in the SA compared to the Ibu (*P* ═ 0.005, 95% CI [−16.83, −2.86]) and Ibu+VPA (*P* < 0.001, 95% CI [5.08, 19.05]) treated groups.

**Figure 3.**
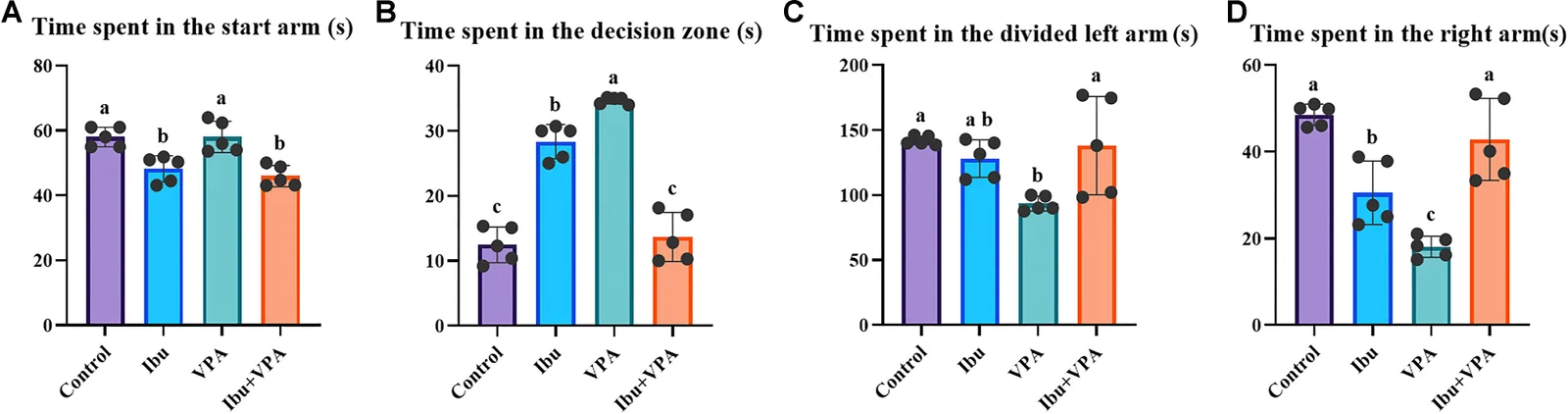
**Time spent in the arms of the T-maze across experimental groups treated with single and combined pharmaceuticals (Ibu = 20 µg L^−1^; VPA = 3 µg L^−1^) over a 96-hour period.** The primary parameters measured include (A) time spent in the start arm (s), (B) time spent in the decision zone (s), (C) time spent in the left divided arm (s), and (D) time spent in the right arm (s). Data are presented as mean ± SD (*n* ═ 5 animals per group) and were analyzed using one-way ANOVA followed by Tukey’s HSD test. Superscript letters (a–c) denote statistically significant differences between groups; identical letters indicate no difference, while different letters signify distinct group means. **Abbreviations:** ANOVA, analysis of variance; HSD, honestly significant difference; Ibu, ibuprofen; SD, standard deviation; VPA, valproic acid.

Regarding the DZ of the T-maze, as shown in Figure 3B, both the Ibu (*P* < 0.001, 95% CI [−20.76, −11.01]) and VPA (*P* < 0.001, 95% CI [−27.09, −17.34]) treated groups exhibited a significant increase in time spent in this zone, while the Ibu+VPA treated group did not show a statistically significant difference from the control group (*P* > 0.05, 95% CI [−6.08, 3.66]). The VPA-treated group spent more time in the DZ compared to Ibu (*P* ═ 0.009, 95% CI [−11.21, −1.45]) and Ibu+VPA groups (*P* < 0.001, 95% CI [16.13, 25.88]). Additionally, the Ibu-treated group spent more time in the DZ than the binary mixture group (*P* < 0.001, 95% CI [9.79, 19.55]).

In the most socially relevant zone of the T-maze (Divided Left Arm), where conspecifics were placed to facilitate visual social interaction, the VPA-treated group exhibited a significant decrease (*P* ═ 0.009, 95% CI [11.49, 85.84]) in time spent in this arm compared to the control group. In contrast, neither the Ibu-treated group (*P* ═ 0.710, 95% CI [−23.22, 51.13]) nor the Ibu+VPA co-exposed group (*P* ═ 0.989, 95% CI [−33.13, 41.21]) displayed statistically significant differences compared to the control group, as depicted in Figure 3C. The VPA-treated group spent significantly less time in the Left Arm compared to the Ibu+VPA treated group (*P* ═ 0.016, 95% CI [−44.62, −7.45]). An opposite trend was observed in the RA of the maze, as shown in Figure 3D. The VPA (*P* < 0.001, 95% CI [19.19, 41.72]) and Ibu (*P* ═ 0.002, 95% CI [6.75, 29.29]) treated groups spent significantly less time than the control group, while the Ibu+VPA treated groups did not exhibit significant alterations in this parameter (*P* ═ 0.491, 95% CI [−5.57, 16.96]). The VPA treated group spent less time in the RA than the Ibu (*P* ═ 0.028, 95% CI [1.16, 23.70]) and Ibu+VPA (*P* < 0.030, 95% CI [−36.03, −13.49]) treated groups. In contrast, the Ibu+VPA treated group spent significantly more time in the RA compared to the Ibu-treated group (*P* ═ 0.030, 95% CI [−23.60, −1.065]).

In addition to the time spent in the T-maze, which is relevant for assessing social preferences, we also evaluated parameters related to general locomotor activity. The analysis of the DT in the T-maze (Figure 4A) revealed that both the Ibu (*P* < 0.001, 95% CI [191.4, 583.6]) and VPA (*p* ═ 0.001, 95% CI [123.7, 515.9]) treatment groups exhibited a significant reduction in locomotor activity compared to the control group. Conversely, the Ibu+VPA treated group did not show significant changes (*P* ═ 0.279, 95% CI [−67.85, 324.3]). The VPA treated group did not demonstrate significant differences compared to either the Ibu (*P* ═ 0.758, 95% CI [−263.8, 128.4]) or the Ibu+VPA (*P* ═ 0.057, 95% CI [−387.6, 4.576]) treated groups. In contrast, the Ibu treated group displayed a significant decrease (*P* < 0.008, 95% CI [−455.3, −62.12]) in DT compared to the Ibu+VPA treated group.

**Figure 4.**
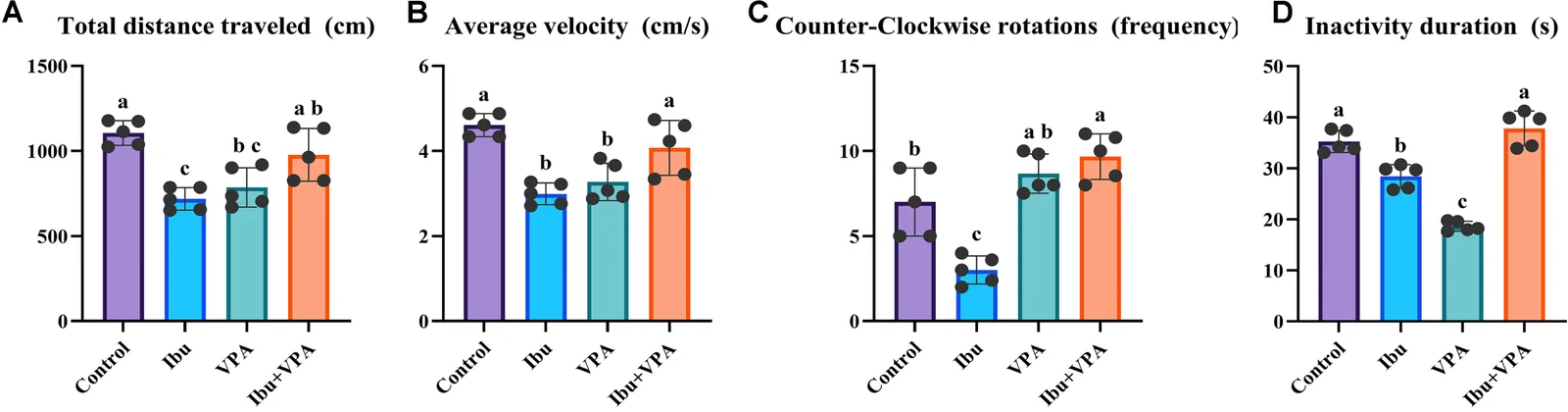
**General parameters of locomotor activity in adult zebrafish treated with single and combined pharmaceuticals (Ibu = 20 µg L^−1^; VPA = 3 µg L^−1^) for 96 h.** The primary parameters assessed include (A) Total distance traveled (cm), (B) Average velocity (cm/s), (C) Duration of inactivity (s), and (D) Frequency of counter-clockwise rotations. Data are presented as mean ± SD (*n* ═ 5 animals per group) and analyzed using one-way ANOVA followed by Tukey’s HSD test. Superscript letters (a–c) indicate statistically significant differences between groups; identical letters denote no difference, while differing letters signify distinct group means. **Abbreviations:** ANOVA, analysis of variance; HSD, honestly significant difference; Ibu, ibuprofen; SD, standard deviation; VPA, valproic acid.

As illustrated in Figure 4B, the AV measured during the single-exposure conditions revealed significant reductions in both the Ibu (*P* < 0.001, 95% CI [0.83, 2.40]) and VPA (*P* < 0.001, 95% CI [0.55, 2.11]) treatment groups compared to the control group. In contrast, the group exposed to the Ibu+VPA mixture did not exhibit statistically significant differences in AV compared to the control group (*p* ═ 0.245, 95% CI [−0.24, 1.32]). The Ibu (*P* < 0.006, 95% CI [−1.08, −0.29]) and VPA (*P* < 0.045, 95% CI [−1.58, −0.01]) treatment groups showed reductions in AV compared to the Ibu+VPA treated group; however, the Ibu treated group did not exhibit significant differences compared to the VPA treated group (*P* ═ 0.735, 95% CI [−1.06, 0.50]).

Regarding other behavioral parameters commonly used in ecotoxicology to assess anxiety or stress-related behaviors, such as ID (Figure 4C) and CCW (Figure 4D), notable treatment effects were observed. For ID, both single-treatment groups demonstrated reductions compared to the control group, with a moderate decrease in the Ibu treated group (*P* ═ 0.02, 95% CI [2.51, 11.07]) and a more pronounced decrease in the VPA treated group (*P* < 0.001, 95% CI [12.32, 20.87]). In contrast, the mixture-treated group did not show significant changes in ID compared to the control group (*P* ═ 0.350, 95% CI [−6.83, 1.71]). The Ibu (*P* < 0.001, 95% CI [−13.63, −5.07]) and VPA (*P* < 0.001, 95% CI [5.52, 14.08]) treated groups exhibited significant decreases compared to the Ibu+VPA treated group. Furthermore, the VPA treated group showed a significant decrease compared to the Ibu treated group (*P* < 0.001, 95% CI [5.52, 14.08]).

Concerning CCW, only the Ibu treated group exhibited a decrease in the frequency of these rotations (*p* ═ 0.002, 95% CI [1.47, 6.52]), while the VPA treated group (*P* ═ 0.273, 95% CI [−4.19, 0.86]) showed a non-significant increase compared to the control group. In contrast, the Ibu+VPA treated group demonstrated an increase in CCW compared to the control group (*P* ═ 0.037, 95% CI [−5.19, −0.13]). The Ibu treated group engaged in fewer CCW rotations than both the VPA *(P <* 0.001, 95% CI [−8.19, −3.13]) and Ibu+VPA (*P* < 0.001, 95% CI [−9.19, −4.13]) treated groups, while the VPA treated group exhibited no significant changes compared to the binary exposure (*P* ═ 0.676, 95% CI [−3.52, 1.52]).

To further elucidate the behavioral patterns within our dataset, we conducted a Pearson correlation analysis. This analysis compared the time spent in each of the three primary maze arms (the SA, the divided left arm, and the RA) with key locomotor parameters (total distance moved, counterclockwise rotations, and immobility duration) (Figure 5A–I). This approach allowed us to assess how spatial occupancy within the T-maze relates to overall activity levels and movement dynamics, offering a more integrated perspective on the interactions among exploratory behavior, social preference, and locomotor performance across treatment groups. Several locomotor and socio-behavioral endpoints exhibited statistically significant relationships under these conditions. Positive correlations were observed between LA (*r* ═ 0.64, *P* < 0.002, 95% CI [0.27, 0.84], *n* ═ 20), RA (*r* ═ 0.83, *P* < 0.0001, 95% CI [0.61, 0.93], *n* ═ 20), and ID. In contrast, a negative correlation was noted between the SA and ID (*r* ═ –0.43, *P* < 0.05, 95% CI [−0.73, 0.01], *n* ═ 20) (Figure 5C).

**Figure 5.**
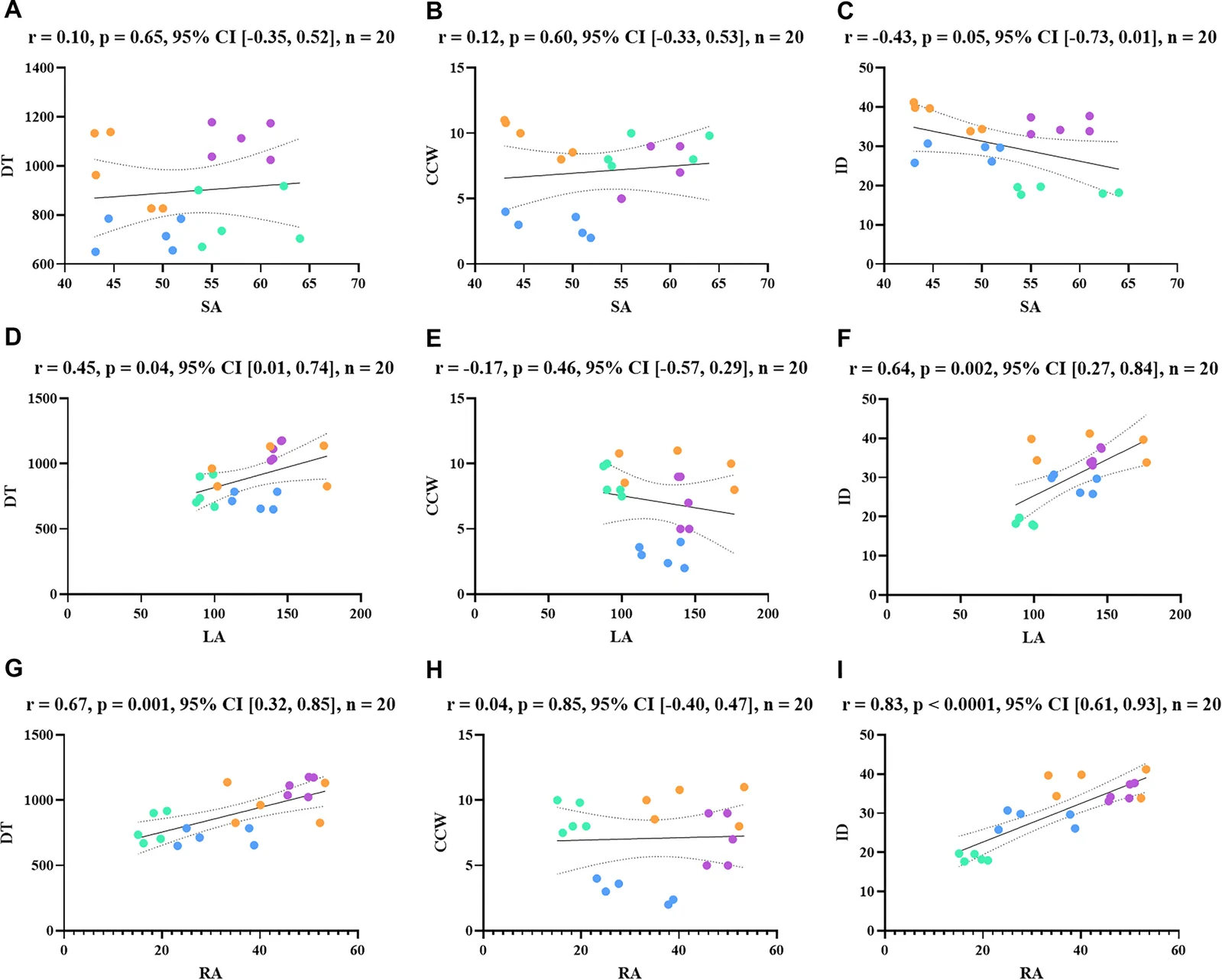
**Pearson’s correlation analysis between social preference and locomotor activity parameters in zebrafish exposed to environmentally relevant pharmaceutical concentrations for 96 h.** (A) SA and DT, (B) SA and CCW, (C) SA and ID, (D) LA and DT, (E) LA and CCW, (F) LA and ID, (G) RA and DT, (H) RA and CCW, (I) RA and ID. The abbreviations are defined as follows: SA denotes time spent in the start arm, LA represents time spent in the divided left arm, RA indicates time spent in the right arm, DT refers to total distance traveled, CCW signifies the frequency of counter-clockwise rotations, and ID pertains to inactivity duration. Data points represent individual zebrafish, with different point colors denoting the Control, Ibu, VPA, and Ibu+VPA groups. The solid line illustrates the fitted linear regression, highlighting the estimated relationship between the two variables. The dashed lines represent the 95% CI bands, indicating the uncertainty surrounding the regression estimate. **Abbreviations**: CCW, counter-clockwise rotations; CI, confidence interval; DT, total distance traveled; Ibu, ibuprofen; ID, inactivity duration; LA, time spent in the divided left arm; RA, time spent in the right arm; SA, time spent in the start arm; VPA, valproic acid.

Overall, no significant associations were observed between CCW and the time spent in SA (*r* ═ 0.12, *P* ═ 0.60, 95% CI [−0.33, 0.53], *n* ═ 20), LA (*r* ═ –0.17, *P* ═ 0.46, 95% CI [−0.57, 0.29], *n* ═ 20), and RA (*r* ═ 0.04, *P ═* 0.85, 95% CI [−0.40, 0.47], *n* ═ 20) (Figure 5B, E, H).

DT exhibited a weak association with SA (*r* ═ 0.10, *P ═* 0.65, 95% CI [−0.35, 0.52], *n* ═ 20), a slightly stronger correlation with LA (*r* ═ 0.45, *P ═* 0.04, 95% CI [0.01, 0.74], *n* ═ 20), and the strongest correlation with RA (*r* ═ 0.67, *P ═* 0.001, 95% CI [0.32, 0.85], *n* ═ 20) (Figure 5A, D, G). In summary, the results highlight a distinct group of locomotor and behavioral variables that exhibited the strongest correlations across exposure conditions, although the limited sample size (*n* ═ 5/group) necessitates cautious interpretation.

## Discussion

This study evaluated the behavioral effects of environmentally relevant concentrations of Ibu and VPA, as well as their combined exposure, in adult zebrafish, utilizing a social preference test alongside locomotor and anxiety-related endpoints. Overall, our findings indicate that 96-hour exposures to a single compound can induce distinct behavioral alterations, whereas combined exposure may result in non-additive effects, underscoring the challenges of predicting the ecological consequences of contaminant mixtures in aquatic systems.

The observed decrease in time spent in the SA for both Ibu- and Ibu+VPA-treated groups suggests a modification in exploratory behavior [57, 58]. The absence of significant effects in the VPA-only group suggests that VPA may not exert a substantial influence on the initial exploratory impulse under the assessed exposure conditions.

Both the Ibu and VPA groups spent increased time in DZ, indicating a potential impairment in decision-making. This finding aligns with the work of Parker et al. (2013), which reported alterations in zebrafish behavior following exposure to substances that disrupt neurotransmission [59]. The unchanged behavior in the Ibu+VPA treated group may reflect compensatory interactions between the Ibu and VPA treatments, resonating with previous research by Backhaus and Faust (2012) [60] suggesting that pharmaceutical mixtures could produce non-additive behavioral effects. Social preference was significantly influenced by VPA exposure, as fish spent less time in LA and more time in the RA, indicating either a reduced preference for social stimuli or a potential manifestation of anxiety-related behavior [61]. This is consistent with prior studies in zebrafish that associate VPA exposure with social deficits due to disruptions in GABAergic signaling and histone deacetylase (HDAC) inhibition [62, 63]. These findings imply that even low levels of VPA may disrupt social behavior, whereas Ibu or combined exposures did not result in significant changes in the time spent in the LA.

Baronio et al. (2018) demonstrated that exposure of zebrafish embryos to VPA leads to significant molecular and neurochemical disturbances that persist into adulthood and are associated with measurable deficits in social behavior [64]. Similarly, Li and Lu (2024) reported a reduction in time spent in the conspecific zone in a zebrafish model of autism, corroborating our observations while also noting increases in both DT and AV [65]. Additionally, Ricarte et al. (2024) found that adults (90 days post-fertilization) derived from embryos exposed to VPA (48 mM) exhibited reduced social engagement in larger shoals and diminished interest in conspecifics [66].

Analysis of locomotor parameters indicates that single exposures to Ibu and VPA may reduce DT and AV, indicating a potential hypoactive response. Islam et al. (2023) demonstrated that Ibu concentrations ranging from 5 to 500 µg L^−1^ induce reduced locomotion in zebrafish larvae (120 hpf), implying that the hypoactivity observed in our study may similarly reflect neurotoxic and enzymatic disorders caused by Ibu [67]. The absence of locomotor impairment in the Ibu + VPA treatment group further supports non-additive interactions between the two compounds. In our experiment, the results regarding social behavior were consistent with those reported by Li and Lu (2024), while the patterns for DT and AV exhibited an opposing trend [65].

Anxiety and stress-related behaviors displayed treatment-specific effects, with decreased ID in groups treated with Ibu or VPA alone, possibly indicating alterations in normal exploratory behavior rather than typical anxiety-like responses [58, 68, 69]. The more pronounced reduction in the VPA group aligns with its recognized anxiolytic properties [70]. Conversely, the exclusive decrease in CCW in the Ibu group suggests an anxiolytic-like effect, as these behaviors are stress-related in zebrafish [71]. The lack of significant effects in the mixture-exposed group may reflect antagonistic interactions between the compounds. The associations identified in this dataset can be elucidated by considering zebrafish locomotion in the T-maze and the interplay between social preference and exploratory drive.

The short duration of the SA suggests that the fish continued typical exploratory behavior rather than avoidance. Increased time spent in LA may be driven by reduced movement or immobility in that zone, rather than a genuine preference for the social stimulus, which would consequently limit exploration in other maze areas. Furthermore, correlations between DT and RA may indicate variations in overall locomotion levels, potentially influenced by stress from pharmaceutical exposure. Research by Firdous et al. (2024) demonstrated that zebrafish with elevated dopamine levels exhibited more intense locomotor activity in open field tests, covering longer distances and moving more rapidly [72].

Decreased locomotion or increased ID may occur in response to reduced stimulation, aligning with minor stress-related alterations. The age-related decline in locomotor activity reported by Evans et al. (2021) may explain the differing behaviors of zebrafish in our experiment compared to those in other studies, a limitation we acknowledge [73].

Additionally, the small sample size (*n* ═ 5 per group) limits the statistical power of our analyses, increasing the likelihood that subtle behavioral effects went undetected. This constraint also diminishes the generalizability of our findings, necessitating cautious interpretation, particularly given the exploratory nature of this investigation. Future studies utilizing larger sample sizes would be better positioned to validate these preliminary observations and could incorporate more robust statistical methods, including nonparametric approaches, to confirm the reliability and reproducibility of the observed effects.

Moreover, the absence of biochemical or molecular confirmation (e.g., neurotransmitter levels, oxidative stress markers, or drug uptake assays) prevents us from validating the mechanistic basis of the observed behavioral changes. Consequently, our conclusions are based solely on behavioral endpoints, which, while informative, do not fully disentangle whether the effects arise from neurochemical alterations, developmental impacts, or other physiological processes. These methodological choices were made in accordance with the 3Rs principles, particularly the Reduction principle, which encourages minimizing the number of animals used while still obtaining meaningful scientific insights. The decision to focus on non-invasive behavioral measures aligns with the Refinement principle by avoiding additional stress or invasive procedures that could compromise animal welfare.

## Conclusion

This study indicates that environmentally relevant levels of Ibu and VPA may influence social behavior, locomotion, and anxiety-related endpoints in zebrafish, with VPA exerting a more pronounced impact on social preferences. Combined exposure to these substances did not exacerbate behavioral changes. Social behavior and locomotion are critical for predator avoidance, foraging efficiency, reproductive success, and overall survival. Disruption of social cohesion may render individuals more vulnerable to predation, impair resource acquisition, or reduce mating opportunities.

Analysis of locomotor parameters reveals that single exposures to Ibu or VPA resulted in significant decreases in DT and AV, indicating hypoactivity. At the population level, such alterations may weaken group dynamics and ultimately compromise the resilience and stability of aquatic communities. Thus, behavioral perturbations related to pharmaceutical exposure may have ecological consequences that extend beyond the individual organism. These findings underscore the necessity of integrating mixture toxicity and behavioral aspects into ecological risk assessments concerning pharmaceutical pollutants in aquatic environments.

## Data Availability

All data generated or analyzed during this study are included in this article. Further inquiries can be directed to the corresponding authors.
